# Does Consolidation of Visuospatial Sequence Knowledge Depend on Eye Movements?

**DOI:** 10.1371/journal.pone.0103421

**Published:** 2014-08-04

**Authors:** Daphné Coomans, Jochen Vandenbossche, Koen Homblé, Eva Van den Bussche, Eric Soetens, Natacha Deroost

**Affiliations:** Department of Experimental and Applied Psychology, Vrije Universiteit Brussel, Brussels, Belgium; University of Bologna, Italy

## Abstract

In the current study, we assessed whether visuospatial sequence knowledge is retained over 24 hours and whether this retention is dependent on the occurrence of eye movements. Participants performed two sessions of a serial reaction time (SRT) task in which they had to manually react to the identity of a target letter pair presented in one of four locations around a fixation cross. When the letter pair ‘XO’ was presented, a left response had to be given, when the letter pair ‘OX’ was presented, a right response was required. In the Eye Movements (EM) condition, eye movements were necessary to perform the task since the fixation cross and the target were separated by at least 9° visual angle. In the No Eye Movements (NEM) condition, on the other hand, eye movements were minimized by keeping the distance from the fixation cross to the target below 1° visual angle and by limiting the stimulus presentation to 100 ms. Since the target identity changed randomly in both conditions, no manual response sequence was present in the task. However, target location was structured according to a deterministic sequence in both the EM and NEM condition. Learning of the target location sequence was determined at the end of the first session and 24 hours after initial learning. Results indicated that the sequence learning effect in the SRT task diminished, yet remained significant, over the 24 hour interval in both conditions. Importantly, the difference in eye movements had no impact on the transfer of sequence knowledge. These results suggest that the retention of visuospatial sequence knowledge occurs alike, irrespective of whether this knowledge is supported by eye movements or not.

## Does Consolidation of Visuospatial Sequence Knowledge Depend on Eye Movements?

Imagine that you take the train to work. This means that, every day, you encounter the same lawns, buildings, and roads. Interestingly, after several subsequent train rides, you acquire knowledge about this sequence of sceneries. For example, you know that the first large building you encounter is a white block of apartments, which is followed by a meadow, and so on. Although such knowledge can be acquired without the intention to learn, little is known about whether this knowledge is consolidated over time. Are you still able to predict the next scenery after your holiday? In the current research, we will determine whether visuospatial sequence knowledge is consolidated over time, and whether this retention is dependent on the occurrence of eye movements.

The acquisition of sequences occurs largely implicit. This means that learning takes place incidentally, without the intention to learn, and with the acquired knowledge being difficult to verbalize [1], [2]. The most popular paradigm to investigate implicit sequence learning is the serial reaction time (SRT) task [3]. In this task, a stimulus appears in one of four locations and participants have to respond to its location with a spatially corresponding key. Unknown to them, this stimulus location is structured according to a predetermined sequence. Results show that participants learn the sequence of locations under these conditions, as indicated by a reaction time (RT) increase when the structured sequence is replaced by a new sequence after sufficient training.

It has been shown that several sequence representations can be formed while performing an SRT task (for a review, see [4]). First, knowledge can be based on the sequence of manual responses, called motor sequence learning [5], [6], [7], [8], [9]. Researchers generally agree that this kind of learning is the most dominant component in sequence learning [5]. However, sequence learning can also be based on the visuospatial movements of the stimulus [10], [11], [12], [13], [14], [15], [16]. Additionally, some alternative accounts suggest that representations of stimulus-response rules [17], [18], [19] and response-effects [20], [21] are built during learning.

Consolidation refers to the preservation or enhancement of knowledge after a time delay. There has already been substantial research on the consolidation of knowledge in a typical SRT task, in which participants manually respond to a sequenced stimulus dimension. In these sequence learning studies, consolidation is consistently reported, either in the form of a preservation or an enhancement of manual motor knowledge [22], [23], [24], [25], [26]. Recently, several researchers have tried to disentangle this consolidation effect by dissociating the different motor components in the SRT task. For example, Cohen et al. [27], [28] dissociated finger effector learning from response location learning by letting participants switch their hands in the experiment: after being trained on a sequence with their right hand, participants had to respond with their left hand in the test phase. In one condition, the presented sequence on the screen and, hence, the response location sequence was maintained, while the effector sequence was changed by the switch of hands. This condition was assumed to reflect goal-based sequence knowledge. In the second condition, the effector sequence remained the same while the response location sequence was altered by presenting a mirrored sequence in the test phase. Interestingly, sequence knowledge based on effectors increased over day, while goal-based sequence knowledge only increased after sleep.

Other studies with an explicit sequence learning task, i.e., the finger tapping task, have yielded similar results with respect to the consolidation of goal-based sequence knowledge. An effector-independent motor representation was maintained by a night of sleep or even a daytime nap, whereas this was not the case for effector-based knowledge. This knowledge only stabilized, irrespective of whether participants slept or not [29], [30].

Thus, different motor sequence components do not seem to consolidate in the same manner. Consequently, results of consolidation studies investigating one type of sequence representation cannot be generalized to other sequence representations. It is therefore necessary to investigate the consolidation of all types of sequence knowledge in order to pronounce upon the role of each component in the formation of permanent sequence knowledge. Studies on the consolidation of visuospatial sequence information are scarce, though. Can sequence knowledge be consolidated at all when it is not supported by a manual sequence? Although goal-based knowledge, as assessed in the studies discussed above [27], [28], [29], [30], cannot be supported by the learning of finger movements, we believe that it is not purely visuospatial in nature either. More specifically, goal-based knowledge may actually refer to response location learning, a learning component that is clearly motor in nature [7], [31].

One study that did investigate the consolidation of visuospatial sequence knowledge is the study of Albouy and colleagues ([32], see also [33]). In this study, sequence learning was not measured by RTs of manual responses, but instead by RTs of oculomotor responses. Participants had to keep track of a target presented in one of four locations on the screen by making an eye movement. The location of the target was structured according to a sequence. In addition, participants also had to make a manual response when the target changed colour, which happened in a minority (20%) of the trials. Only a visuospatial, but not a sequence of manual responses, could be learned. The results of this study showed that visuospatial sequence knowledge supported by oculomotor movements is consolidated over time, as sequence knowledge was maintained for 30 minutes and 5 hours, but increased after 24 hours.

However, visuospatial sequence knowledge in this latter study was supported by eye movements. The current research was designed to investigate whether visuospatial sequence knowledge that is not supported by eye movements can also be consolidated. To this end, participants performed two sessions, with an interval of 24 hours, of an adapted SRT task in which a target letter pair (“XO” or “OX”) appeared in one of four locations on the screen (see also [13]). Participants had to respond to the identity of the target letter pair, which changed randomly. When the letter pair “XO” was presented, a left response had to be given, when the letter pair “OX” was presented, a right response was required. Unknown to participants, the location of the target was structured according to a deterministic sequence. In the ‘No Eye Movements (NEM)’ condition, eye movements were minimized by presenting the letter pairs very close to a fixation cross and limiting the stimulus presentation time to 100 ms (see [10]). In the ‘Eye Movements (EM)’ condition, on the other hand, letter pairs were widely separated so that participants were required to make oculomotor movements to be able to respond to the target. We expect that visuospatial sequence knowledge supported by eye movements will be preserved or enhanced over time, as was found in the study of Albouy et al. [32]. However, it is not clear whether visuospatial sequence knowledge that is not supported by eye movements will also consolidate over the 24 hour interval.

## Method

### Ethics Statement

The experimental procedures were executed in compliance with local laws and institutional guidelines. Based on our protocol, the Medical Ethics Committee of the Vrije Universiteit Brussel decided that our study was exempt from approval (reference 2013/086). The subjects were all students of the Vrije Universiteit Brussel, who participated in the experiment in return for course credit. A written informed consent was obtained from all participants.

### Participants

In order to determine consolidation of sequence knowledge, only participants showing a reliable learning effect of at least 10 ms at the end of Session 1 were included in the study (as a consequence, 25% of the participants were omitted). From the resulting participants, 12 participants (2 men, mean age  = 18.83, *SD*  = 1.11) completed the experiment in the condition where eye movements were minimized (NEM condition) and 12 participants (5 men, mean age  = 19.08, *SD*  = 0.90) completed it in the condition where eye movements were more substantial (EM condition). Participants were tested at hours ranging from 10 a.m. to 3 p.m., with an interval of exact 24 hours between the beginning of Session 1 and the beginning of Session 2 for each participant.

The data of Session 1 of participants in the NEM condition have already been used in a previous paper of our laboratory on the relationship between perceptual sequence learning and eye movements [10]. In the current paper, we build upon this work by assessing whether this kind of knowledge can be consolidated.

### Stimuli and apparatus

The experiment was programmed and run in the software program E-Prime 2 Professional [34]. Participants executed the two sessions of the experiment in semi-darkened cubicles of the psychological laboratory of the Vrije Universiteit Brussel on Pentium 4 personal computers with 17-inch CRT monitors.

**Figure 1:**
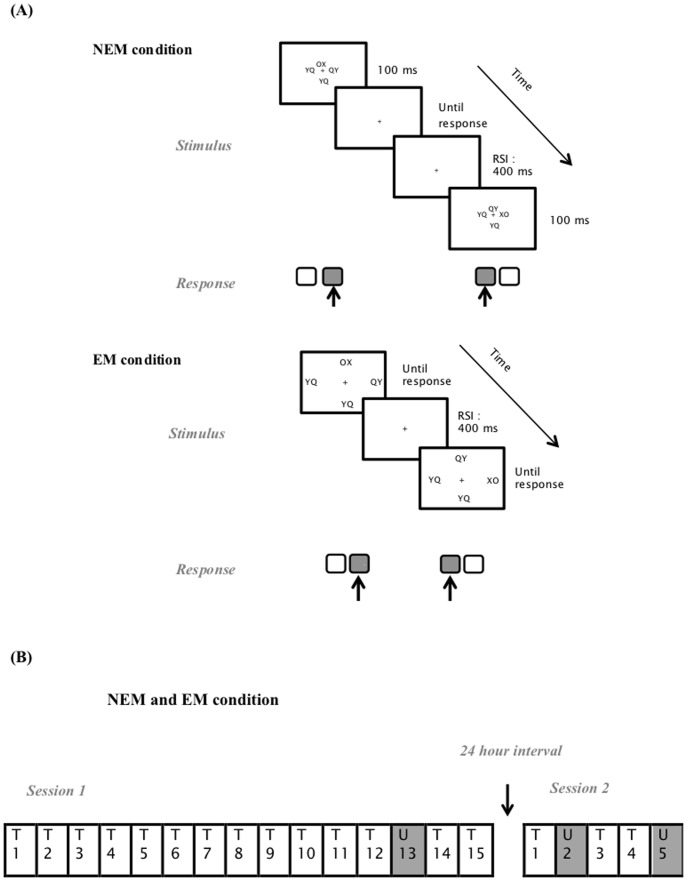
Research method. (A) Example of two consecutive trials in the no eye movements (NEM) and the eye movements (EM) condition. Participants had to respond to the identity of the target letter pair “XO” or “OX”; “XO” required a left response, “OX” a right one. In the NEM condition, target and distractors (“YQ” or “QY”) were presented close to the fixation cross and were displayed for 100 ms, after which only the fixation cross remained on the screen. The next trial started 400 ms (RSI) after a response was given. In the EM condition, target and distractors were widely separated and remained on the screen until participants responded. In both conditions, target identity, and hence manual responses, changed randomly, while target location was structured according to a deterministic sequence. In the current example, target location 1 (above the fixation cross) was followed by target location 2 (right from the fixation cross). (B) Overview of the design, which was similar in the NEM and the EM condition. T  =  trained blocks, U  =  untrained blocks.

In both conditions, a letter pair was presented above, right, below and left from a fixation cross in black against a white background (see Figure 1a for an overview of a trial procedure in the NEM and the EM condition). One letter pair was the target letter pair “XO” or “OX”, the remaining locations were filled with the distractor letter pairs “YQ” and “QY”. In the NEM condition, letter pairs were displayed in Arial point size 6 and measured 0.5 cm width × 0.3 cm height. The distance between the centre of the fixation cross and the centre of the letter pairs was 0.6 cm (corresponding to a visual angle of 0.63°) at a viewing distance of approximately 55 cm. In the EM condition, letter pairs were presented in a larger Arial point size 9 and measured 0.7 cm width × 0.5 cm height. The distance between the centre of the fixation cross and the centre of the letter pairs was 9 cm or 9.29° vertically and 12.2 cm or 12.51° horizontally.

### Procedure

#### SRT task

Participants were asked to respond as quickly and accurately as possible to the identity of the target letter pair “XO” or “OX”. The target “XO” required a ‘C’-response with the left index finger, the target “OX” an ‘N’-response with the right index finger. In case of an erroneous response, or no response within 3000 ms, an error message was displayed in Dutch for 750 ms. After a response-stimulus interval of 400 ms the next trial was presented.

In order to hinder or stimulate oculomotor movements in the NEM and the EM condition, respectively, there were three main differences between both conditions: (1) while participants in the NEM condition were urged to focus on the fixation cross without making eye movements, participants in the EM condition were not, (2) the visual angle used to display the letter pairs was small (<1° visual angle) in the NEM condition and large (>9° visual angle) in the EM condition, and (3) letter pairs were presented for 100 ms in the NEM condition, after which only the fixation cross remained on the screen, whereas stimulus duration was not limited in the EM condition. In this latter condition, stimuli remained on the screen until a response was given or until the response time of 3000 ms elapsed.

In both conditions, the experiment consisted of two sessions separated by 24 hours. Session 1 began with two practice blocks of 50 trials. In these blocks, the location of the target changed pseudorandomly, with immediate location repetitions being excluded. The target and three distractor identities always varied randomly and individually per trial, with an equal probability of each target letter pair (“XO” or “OX”) and distractor letter pair (“YQ” or “QY”). After each block, participants received feedback about their error rates and RTs, followed by a break of 30 seconds.

After practice, 15 experimental blocks of 100 trials were presented. In these blocks, target location was structured according to a sequence (S1 or S2; S1 and S2 were evenly distributed over the NEM and the EM condition) in all blocks but Block 13. The sequences, adopted from [32], were 42132431 (S1) or 13423124 (S2) with 1 referring to the location above the fixation cross and the remaining numbers to the locations in a clockwise manner. In Block 13, an untrained sequence (S2 or S1) with the same structural properties as the trained sequence was placed on the target's location to assess sequence learning. Session 2, administered 24 hours after Session 1, consisted of 5 blocks of 100 trials. In Blocks 1, 3 and 4, the sequence trained in Session 1 was imposed on the target's location. In Blocks 2 and 5, the untrained sequence was placed on the target's location to determine sequence learning in Session 2. Untrained Block 5 was inserted in case Block 2 proved to be insensitive to consolidation effects, as additional training might be required to reactivate sequence knowledge after a time interval of 24 hours. An overview of the design can be found in Figure 1b.

#### Awareness and sleep

Awareness of the sequence knowledge was assessed after the computer experiment of Session 2 by means of a generation task, based on the process dissociation procedure (PDP) [35], [36]. Participants were presented 16 trials on a sheet of paper. Per trial, four letter pairs (all “OX”) were displayed around a fixation cross (above, right, below and left from the cross). First, participants performed the task under inclusion instructions, which means that they were asked to mark a sequence of 16 locations that they believed occurred frequently in the experiment (with the restriction that no immediate repetitions could be reported). For example, if they thought that the target moved from the bottom to the top, and then to the location right from the cross, they marked the bottom location in the first trial, the top location in the second trial, and the right location in the third trial. Secondly, participants performed the task under exclusion instructions. Now, participants had to mark the transitions that were not frequently presented in the experiment. According to the PDP logic, participants should be able to report more correct transitions under inclusion than under exclusion instructions if they are consciously aware of the location sequence, because this would mean that they are able to control their sequence knowledge under exclusion instructions.

Finally, because previous research has shown that consolidation of sequence knowledge might be mediated by sleep (e.g., [23], [30]), sleep characteristics over night between Session 1 and Session 2 were assessed. Participants were asked to answer the following questions on a sheet of paper before starting the experiment in Session 2. First, they were asked how many hours they slept during the last night. Second, subjective sleep quality was determined by letting the participants (a) assign a number to their sleep quality from 0 to 10 (0 =  extremely bad; 10 =  extremely good) and (b) to the extent to which they felt rested (0 =  completely exhausted; 10 =  completely rested).

## Results

The first trial of each block was discarded from the analyses. Erroneous responses were excluded from the RT analyses. The latter measure resulted in a data loss of 12.65% in the NEM condition and 2.25% in the EM condition. When the assumption of sphericity was not fulfilled, the Greenhouse-Geisser correction is reported. Because the error rates did not always fulfil the assumptions underlying an ANOVA, analyses on error rates were conducted on log10 transformed data.

### General training effect in Session 1

A general training effect was determined by a 12 × 2 mixed ANOVA, with Block (the first 12 blocks of Session 1) as within-subjects factor and Condition (NEM versus EM) as between-subjects factor to assess whether participants' performance improved during training of the sequence.

#### Reaction times

**Figure 2:**
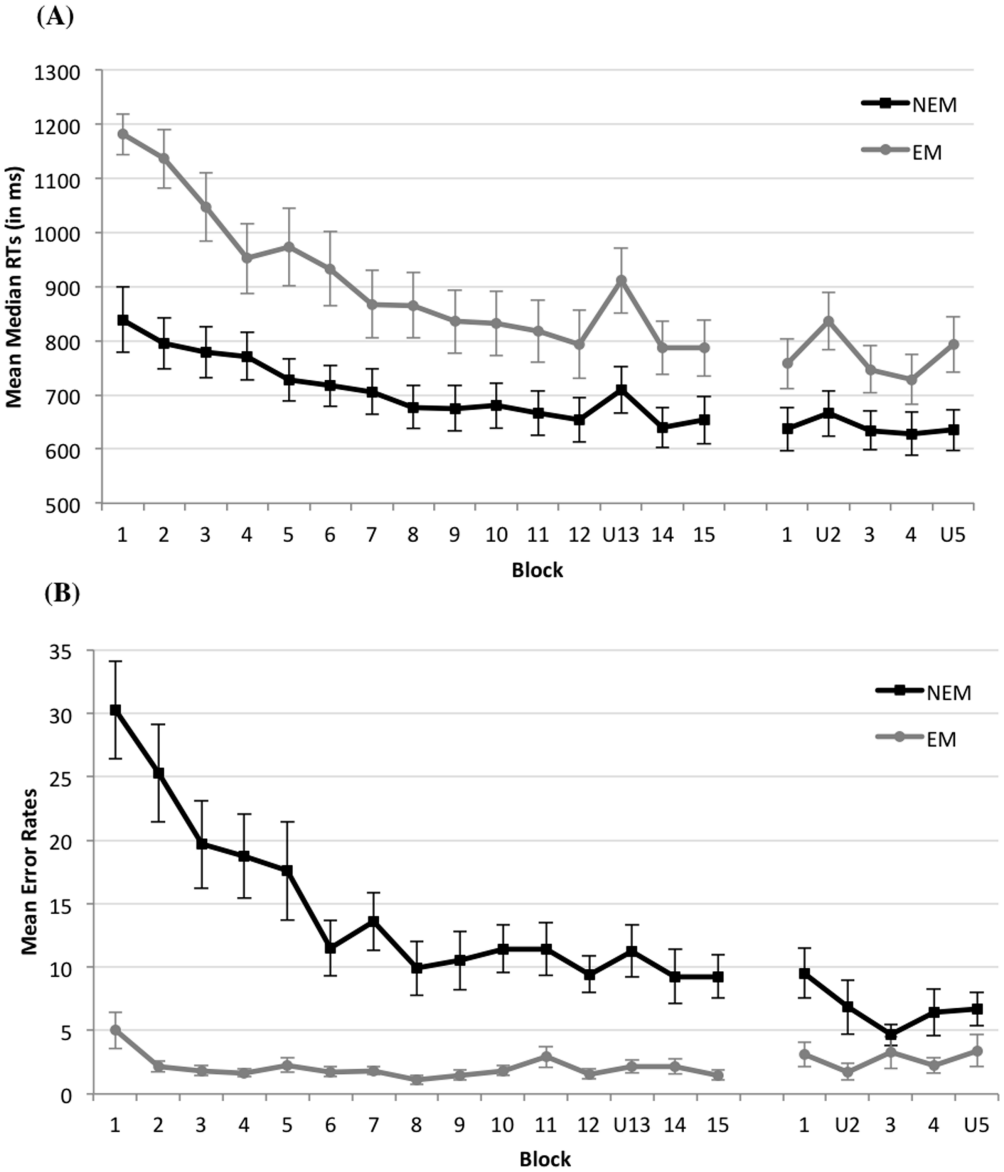
Performance per block for the no eye movements (NEM) and eye movements (EM) condition. In panel (A), mean median reaction times are presented. In panel (B), mean (untransformed) error rates are presented. In Session 1, the trained sequence changed to an untrained sequence in Block 13 (U13). In Session 2, the trained sequence changed to an untrained sequence in Blocks 2 (U2) and 5 (U5). Error bars denote standard errors of the mean. Note: some of the data (i.e., the data of the first session of the participants in the NEM condition) were included in our paper previously published in Experimental Psychology [10].

Figure 2a provides an overview of the mean median RTs per block for the 2 conditions. There was a main effect of Condition, reflecting higher RTs in the EM as compared to the NEM condition, *F*(1,22)  = 9.09, *MSE*  = 356484, *p* = .006, η_p_^2^  = .29. A main effect of Block indicated that RTs decreased over training, *F*(3,68)  = 49.68, *MSE*  = 14817, *p*<.001, η_p_^2^  = .69. There was also a Block by Condition interaction, suggesting that this decrease was larger in the EM than in the NEM condition, *F*(3,68)  = 7.53, *MSE*  = 14817, *p*<.001, η_p_^2^  = .25.

#### Error rates

Figure 2b provides an overview of the mean (untransformed) error rates per block for the 2 conditions. The ANOVA on log10 transformed error rates yielded a main effect of Condition, *F*(1,22)  = 60.74, *MSE*  = 0.58, *p*<.001, η_p_^2^  = .73. A main effect of Block indicated that error rates decreased during training, *F*(11,242)  = 12.20, *MSE*  = 0.031, *p*<.001, η_p_^2^  = .36, but a Block by Condition interaction showed that this decrease was more pronounced in the NEM condition, *F*(11,242)  = 2.58, *MSE*  = 0.031, *p* = .0041, η_p_^2^  = .10. These results indicate that the task in the NEM condition was more difficult than in the EM condition: participants in the NEM condition made more errors at the start of the experiment, leaving more room for improvement over training compared to the participants in the EM condition.

### Sequence learning in Session 1

Subsequently, we determined whether participants had acquired sequence-specific knowledge at the end of Session 1 in both conditions. This was done by means of a 2 (Sequence learning: untrained Block 13 versus the mean of the adjacent trained Blocks 12 and 14) × 2 (Condition: NEM versus EM) mixed ANOVA. If participants learned the sequence, they should react slower (or with more errors) in untrained Block 13 than in the adjacent trained Blocks 12 and 14. An overview of the mean learning effects, i.e. the difference in mean median RTs and error rates between the untrained block and the adjacent trained blocks, can be found in Table 1.

**Table 1:** Overview of sequence learning effects with their standard deviation (SD) per condition and per session.

Condition	Session	RTs (in ms)	Error rates	Log10 transformed error rates
**NEM**	Session 1	63 (39.9)	1.92 (3.33)	0.053 (0.18)
	Session 2	30 (42.9)	−0.25 (4.97)	−0.11 (0.32)
**EM**	Session 1	121 (76.8)	0.29 (1.64)	0.051 (0.28)
	Session 2	84 (78.7)	−1.42 (1.69)	−0.18 (0.20)

*Note.* Learning effects were calculated by subtracting performance in the trained blocks from performance in the untrained blocks. For Session 1, the average of Blocks 12 and 14 was subtracted from Block 13; for Session 2, the average of Blocks 1 and 3 was subtracted from Block 2. NEM  =  no eye movements, EM  =  eye movements.

#### Reaction times

The ANOVA on the mean median RTs demonstrated a main effect of Condition, indicating that RTs were higher in the EM than in the NEM condition, *F*(1,22)  = 6.14, *MSE*  = 58129, *p* = .021, η_p_^2^  = .22. There was also a main effect of Sequence learning, *F*(1,22)  = 54.21, *MSE*  = 1873, *p*<.001, η_p_^2^  = .71. The Sequence learning by Condition interaction indicated that participants in the EM condition showed more learning than participants in the NEM condition, *F*(1,22)  = 5.44, *MSE*  = 1873, *p* = .029, η_p_^2^  = .20. However, planned comparisons revealed that sequence-specific learning was present in both conditions, *F*(1,22)  = 12.65, *MSE*  = 1873, *p*  = .0018 for the NEM condition and *F*(1,22)  = 47.01, *MSE*  = 1873, *p*<.001 for the EM condition. Thus, as expected, participants in both conditions displayed sequence knowledge at the end of Session 1.

#### Error rates

The ANOVA on log10 transformed error rates demonstrated a main effect of Condition, indicating that error rates were higher in the NEM condition, *F*(1,22)  = 32.73, *MSE*  = 0.12, *p*<.001, η_p_^2^  = .60. No other effects were significant (both *p*>.29).

### Comparison sequence learning Session 1 versus sequence learning Session 2

To determine whether knowledge obtained in Session 1 was consolidated over 24 hours, a 2 × 2 × 2 mixed ANOVA was run with Session (Session 1 versus Session 2) and Sequence learning (untrained Block 13 versus the average of trained Blocks 12 and 14 for Session 1 and untrained Block 2 versus the average of trained Blocks 1 and 3 for Session 2) as within-subjects factors and Condition (NEM versus EM) as between-subjects factor. Untrained Block 2 of Session 2 was chosen as a measure of consolidation. This way, the possibility that consolidation effects in fact result from relearning is minimized because participants only encountered one sequenced block before untrained Block 2 in Session 2. Learning effects per session in RTs and error rates are presented in Table 1.

#### Reaction times

Participants in the EM condition responded slower than participants in the NEM condition, as indicated by a main effect of Condition, *F*(1,22)  = 5.91, *MSE*  = 101512, *p* = .024, η_p_^2^  = .21. A main effect of Session indicated that RTs decreased from Session 1 to Session 2, *F*(1,22)  = 20.00, *MSE*  = 2090, *p*<.001, η_p_^2^  = .48. This reduction was similar for both conditions, as there was no Session × Condition interaction, *F*(1,22)  = 2.39, *MSE*  = 2090, *p* = .14, η_p_^2^  = .10. The ANOVA also demonstrated a main effect of Sequence learning, *F*(1,22)  = 47.32, *MSE*  = 2815, *p*<.001, η_p_^2^  = .68. This learning effect was larger in the EM condition than in the NEM condition, as can be derived from the Sequence learning by Condition interaction, *F*(1,22)  = 6.77, *MSE*  = 2815, *p* = .016, η_p_^2^  = .24. The most important effect was the significant Session by Sequence learning interaction, which indicated that the learning effect, thus the difference between the trained and untrained blocks, decreased from Session 1 to Session 2, *F*(1,22)  = 6.88, *MSE*  = 1066, *p* = .016, η_p_^2^  = .24 (see Figure 2a). This evolution was present in both conditions, as the three-way interaction did not approach significance, *F*<1. Planned contrasts indicated that sequence knowledge was significant in both sessions though, *F*(1,22)  = 54.21, *MSE*  = 1873, *p*<.001 in Session 1 and *F*(1,22)  = 19.43, *MSE*  = 2008, *p*<.001 in Session 2.

**Figure 3:**
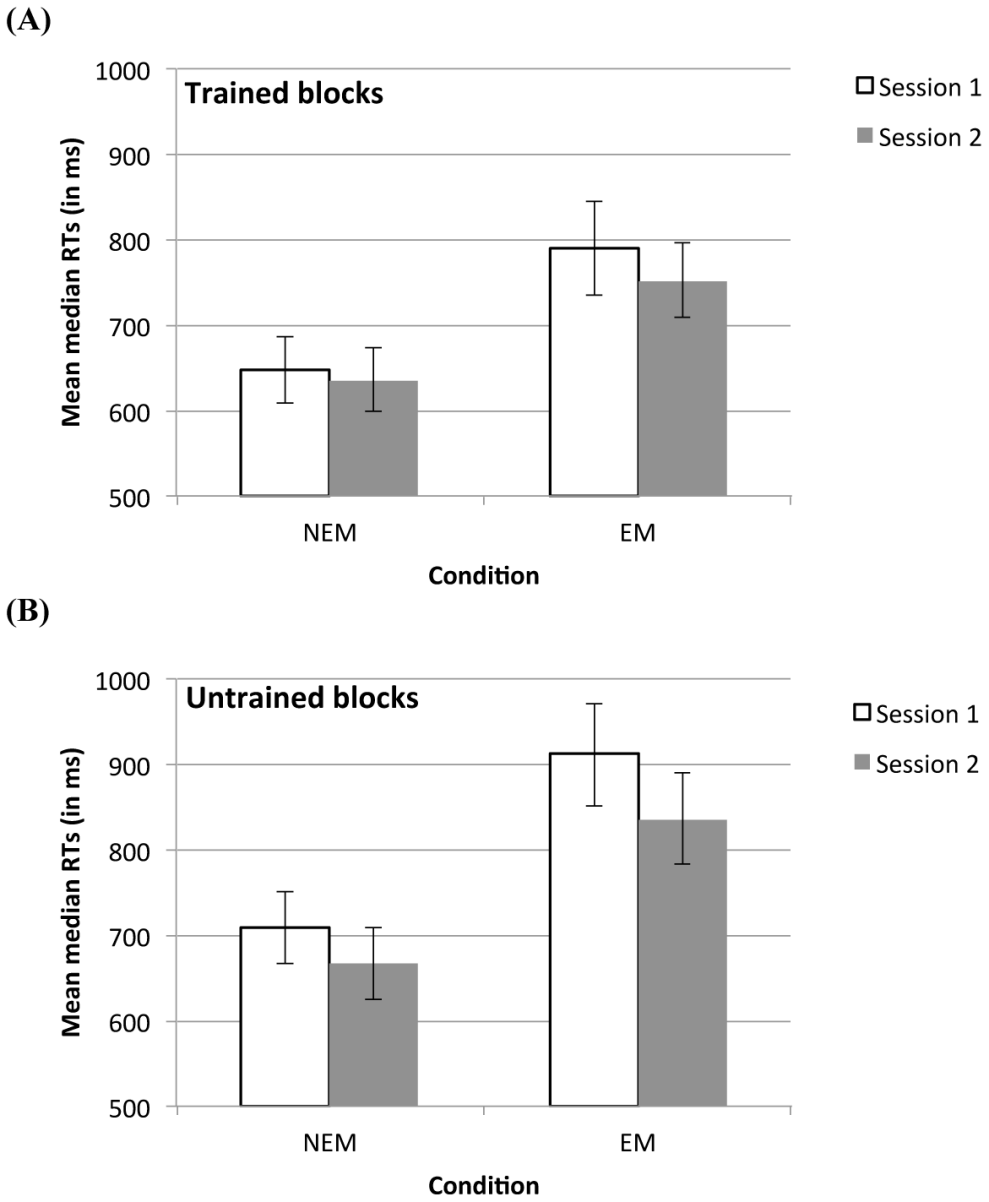
Reaction time (RT) performance on trained and untrained blocks in Session 1 and 2 for the no eye movements (NEM) and the eye movements (EM) condition. (A) Mean median RTs for trained Blocks 12-14 of Session 1 and trained Blocks 1-3 of Session 2 per condition. (B) Mean median RTs for untrained Block 13 of Session 1 and untrained Block 2 of Session 2 for both conditions. Error bars denote standard errors of the mean.

In order to verify whether the smaller learning effect in Session 2 compared to Session 1 was primarily due to a different performance on the trained blocks, the untrained blocks, or both, planned contrasts on the Session by Sequence learning interaction were performed. Consolidation of sequence-specific knowledge would be derived from a better performance on the trained blocks while the performance on untrained blocks remains unchanged [32]. However, because the sequence-specific learning effect was smaller in Session 2 than in Session 1, it is unlikely that our further analyses will reveal this kind of pattern. The planned contrasts indicated that both the RTs on the trained blocks and the RTs on the untrained blocks improved over sessions, *F*(1,22)  = 4.88, *MSE*  = 1446, *p* = .038 and *F*(1,22)  = 24.60, *MSE*  = 1710, *p*<.001, respectively. Figure 3 illustrates the RT performance on trained and untrained blocks in Session 1 and 2 per condition.

Consequently, although both the RTs on the trained and untrained blocks decreased over sessions, this decline was larger for the untrained blocks. The latter can be derived from the main analysis, which indicated that the sequence learning effect (calculated as the difference between untrained and trained blocks) was larger in Session 1 than in Session 2.

#### Error rates

A mixed ANOVA with Session and Sequence learning as within-subjects factors and Condition as between-subjects factor was also performed on log10 transformed error rates. This analysis yielded a main effect of Condition, *F*(1,22)  = 19.67, *MSE*  = 0.26, *p*<.001, η_p_^2^  = .47, indicating that more errors were made in the NEM as compared to the EM condition. There was a main effect of Session, *F*(1,22)  = 6.78, *MSE*  = 0.038, *p* = .016, η_p_^2^  = .24, but a Session by Condition interaction indicated that only in the NEM condition, error rates decreased over sessions, *F*(1,22)  = 7.37, *MSE*  = 0.038, *p* = .013, η_p_^2^  = .25. There was no main effect of Sequence learning, nor was there a Sequence learning × Condition interaction, *F*(1,22)  = 1.42, *MSE*  = .036, *p* = .25, η_p_^2^  = .061 and *F*<1, respectively. There was, however, a Session × Sequence learning interaction, *F*(1, 22)  = 8.41, *MSE*  = 0.027, *p* = .0083, η_p_^2^  = .28. To assess sequence learning in both sessions separately, planned comparison tests were run. These results revealed that sequence learning was not significant in Session 1, *F*(1,22)  = 1.14, *MSE*  = 0.028, *p* = .30, but was significant in Session 2, *F*(1,22)  = 7.15, *MSE*  = 0.035, *p* = .014. In Session 2, however, learning effects were negative (see Table 1 and Figure 2b). This might indicate that the learning effects in Session 2 observed in the RT analysis may partly be accounted for by a speed-accuracy trade-off (SAT). However, because the overall difference in error rates between the untrained and trained blocks of Session 2 was small, namely 0.25 in the NEM condition and 1.42 on a total of 100 trials per block in the EM condition, it seems that learning effects in RTs cannot entirely be explained by an SAT. Finally, there was no three-way interaction, *F* <1, which indicates that consolidation of sequence knowledge did not differ between the NEM and the EM condition.

### Sequence knowledge towards the end of Session 2: trained Block 4 versus untrained Block 5

So far, we only looked at the sequence learning effect at the beginning of Session 2. However, Figure 2a shows that there might be a difference between the two conditions in the use of the sequence knowledge at the end of Session 2. To investigate this effect, a 2 (Sequence learning: trained Block 4 versus untrained Block 5) × 2 (Condition: NEM versus EM) mixed ANOVA was performed.

#### Reaction times

The analysis indicated a main effect of Condition, *F*(1,22)  = 4.46, *MSE*  = 44935, *p* = .046, η_p_^2^  = .17, suggesting that RTs were higher in the EM as compared to the NEM condition. There was also a main effect of Sequence learning, *F*(1, 22)  = 8.99, *MSE*  = 1699, *p* = .0066, η_p_^2^  = .29, as well as a Sequence learning by Condition interaction, *F*(1,22)  = 5.89, *MSE*  = 1699, *p* = .024, η_p_^2^  = .21. A post hoc test with Bonferroni correction revealed that RTs in untrained Block 5 were higher than in trained Block 4 in the EM condition (*p* = .0054), but not in the NEM condition (*p* = 1). Sequence-specific learning, however, was present at the beginning of Session 2 in both conditions, as indicated by paired-samples *t*-tests comparing the RTs of untrained Block 2 with the average RTs of trained Blocks 1 and 3 for each condition separately, *t*(11)  = 2.41, *p* = .035 for the NEM condition and *t*(11)  = 3.71, *p* = .0035 for the EM condition.

#### Error rates

The same ANOVA performed on log10 transformed error rates demonstrated a main effect of Condition, *F*(1,22)  = 5.65, *MSE*  = 0.20, *p* = .027, η_p_^2^  = .20, suggesting that error rates were higher in the NEM condition. Error rates tended to be higher in untrained Block 5 than in trained Block 4, as indicated by a tendency towards a main effect of Sequence learning, *F*(1,22)  = 3.94, *MSE*  = 0.024, *p* = .060, η_p_^2^  = .15. There was no Sequence learning × Condition interaction, *F*<1.

### Generation task

To determine sequence awareness, the reproduction of triplets in the generation task was analysed. Participants were asked to produce a sequence of 16 elements, so a total of 14 triplets were generated under both inclusion and exclusion instructions. Because there were 36 possible triplets to be generated (immediate repetitions were not allowed and not analysed if they occurred nonetheless) of which 8 were correct, chance level was .22.

According to the PDP logic, participants should be able to report more correct transitions under inclusion than under exclusion instructions if they are consciously aware of the location sequence. In both the NEM and the EM condition, inclusion but not exclusion scores differed significantly from chance level (see Table 2). The difference between implicit and explicit instructions was assessed by a 2 (Instruction: inclusion versus exclusion score) × 2 (Condition: NEM versus EM) mixed ANOVA, which demonstrated no main effect of Condition, *F*(1,22)  = 2.44, *MSE*  = .036, *p* = .13, η_p_^2^  = .10. However, participants produced more correct triplets under inclusion than under exclusion instructions, as indicated by a main effect of Instruction, *F*(1,22)  = 7.74, *MSE*  = .047, *p* = .011, η_p_^2^  = .26. This effect was not influenced by Condition, as shown by an absent Instruction by Condition interaction, *F*<1. Thus, participants had at least some comparable amount of explicit knowledge about the sequence in both conditions, as they were able to control their knowledge under exclusion instructions.

**Table 2:** Generation scores and their standard deviation (SD) per condition.

Condition	Inclusion	Exclusion
**NEM**	.34 (.17)*	.19 (.19)
**EM**	.45 (.25)*	.25 (.19)

Note. NEM  =  no eye movements; EM  =  eye movements. * Significantly different from chance at .05 level.

### Sleep questionnaire

In the NEM condition, participants slept on average 7.48 hours (*SD*  = 1.35), rated their sleep quality 7.67 (*SD*  = .89) and the extent to which they felt rested 6.75 (*SD*  = 1.14). Participants in the EM condition slept on average 6.83 hours (*SD*  = 1.53), rated their sleep quality 6.92 (*SD*  = 1.98) and the extent to which they felt rested 5.88 (*SD*  = 1.84). A Mann-Whitney test was performed to determine whether the experimental conditions differed with respect to sleep characteristics. However, no significant differences between the NEM and EM condition could be observed, *U*(24)  = 59, *p* = .48 (hours sleep); *U*(24)  = 50, *p* = .22 (sleep quality) and *U*(24)  = 43, *p* = .10 (extent to which they felt rested).

## Discussion

In the current study, we examined the consolidation of visuospatial sequence knowledge that was not supported by eye movements (NEM condition) and compared this to the consolidation of visuospatial sequence knowledge supported by eye movements (EM condition). Participants performed a modified version of the SRT task in which they had to react to the identity of a target letter pair, while its location was structured. However, whereas oculomotor movements were necessary to locate the target in the EM condition, they were minimized in the NEM condition. Our results revealed a smaller sequence learning effect expressed in the SRT task after a 24 hour interval in both the EM and NEM condition, although learning remained significant in both conditions after the time delay. Importantly, no difference in retention was observed between visuospatial sequence knowledge supported by eye movements and visuospatial sequence knowledge that was not supported by eye movements. Participants in both conditions also displayed explicit awareness after the experiment, but the amount of explicit knowledge did not differ between the EM and NEM condition.

Consolidation of sequence knowledge refers to a preservation or enhancement of sequence-specific knowledge after a time delay. In the current study, the sequence-specific learning effect was always smaller in Session 2 than in Session 1. Further analyses on trained and untrained blocks separately revealed that RTs on both trained and untrained blocks decreased over the time interval. However, the smaller learning effect in Session 2 as compared to Session 1 was due to the larger decrease in RTs on untrained blocks than on trained blocks. Thus, the effect was unrelated to the learning of the sequence itself as sequence-specific knowledge does not induce faster RTs in untrained blocks. Faster RTs to both trained and untrained blocks over the interval are thus likely not indicative for consolidation of the sequence knowledge, but are probably more a reflection of familiarity with sequence-unrelated task aspects, such as stimulus detection and discrimination processes or stimulus-response mapping processes (see also [32]). Additionally, the build-up of fatigue may also have impaired performance at the end of Session 1, while participants began with renewed energy after the interval, leading to faster RTs at the beginning of Session 2 (see [37]). We therefore conclude that sequence knowledge was at best not fully consolidated, because the knowledge was not fully preserved or enhanced over the 24 hour interval. However, although the sequence learning effect was smaller after the interval, not all sequential knowledge seemed to be forgotten as sequence learning was still present at the beginning of Session 2 in both conditions. In sum, visuospatial sequence knowledge was (at best) only partially consolidated, irrespective of whether this knowledge was supported by eye movements or not.

A parallel can be drawn between our study and the traditional perceptual/motor distinction in the sequence learning research field. In the current research, the visuospatial sequence was supported by an oculomotor sequence in the EM condition. Hence, learning in this latter condition may be regarded as a form of motor sequence learning. In our NEM condition, on the other hand, all corresponding motor responses were avoided. Therefore learning in this condition may be comparable to perceptual sequence learning. According to our knowledge, only one study previously investigated whether perceptual sequence knowledge can be consolidated. In the study of Hallgató and collagues [38], participants had to respond to the direction of an arrow presented in the centre of the screen. In the initial training phase, they had to mentally rotate the arrow with 90° and answer with the response key corresponding to this latter direction. Unknown to participants, the direction of the arrow, and hence also the responses to the arrow, followed a regular sequence. After a time delay of 12 or 24 hours, participants continued to respond to the direction of the arrow, but this time by using a different stimulus-response mapping. More specifically, they did not have to rotate the arrow any longer, but now had to respond with the key directly corresponding to its direction. The authors made sure that in the perceptual condition, the presented sequence remained the same but the response sequence changed due to the mapping change, whereas in the motor condition the perceptual sequence changed so that the motor sequence was preserved. The results indicated that perceptual sequence knowledge transferred less than motor sequence knowledge, but it was still significantly present after a delay of 12 and 24 hours. Moreover, in none of the conditions, consolidation of the knowledge was dependent on whether participants had slept during the interval or not. However, two factors have to be taken into account. First, the authors did not determine perceptual sequence knowledge before the time interval (the change in mapping was only introduced after the delay), so it is impossible to establish whether knowledge actually declined over the interval or was already less significant than motor sequence knowledge after initial training. Second, because the motor sequence only shifted one response key after transfer, participants could have displayed a learning effect in the second session based on a simple abstraction of motor sequence knowledge (same response plus counter clockwise shift) instead of on pure perceptual sequence knowledge. Consequently, what the authors denoted as perceptual sequence knowledge could actually be reflecting response-shifted motor knowledge. Nonetheless, our results reveal that even when those factors are controlled for by testing for sequence knowledge before the delay and by using a random manual response sequence, like was done in the current study, the perceptual learning effect still diminishes after a time delay of 24 hours.

The result that visuospatial sequence knowledge supported by eye movements, which may be regarded as a form of motor learning, decreased over the interval, was rather surprising. We hypothesized that this kind of knowledge would be consolidated over time, as previous research showed that oculomotor sequence knowledge increases after a time delay of 24 hours [32]. Yet, a crucial difference between the study of Albouy et al. and our study is that the integration of structured information was likely easier to accomplish in their study, as less noise was interspersed between trials: participants in their study had to respond manually to a colour change of the target, which only happened in 20% of the trials. In contrast, in our task, participants had to manually respond to the identity of a randomly changing target on *each* trial. Thus, compared to the study of Albouy et al., more noise was present on the manual response level in our study because of the random manual responses made on every trial, which might have interfered with the consolidation of the knowledge.

Several measures were taken to constrain eye movements in the NEM condition, including the short presentation of the target and distractor letter pairs during 100 ms. This manipulation, however, not only restricted eye movements in the NEM condition, but also made the task harder to perform than in the EM condition, where no presentation limit was used. This was reflected in higher error rates in the former condition. One might therefore wonder whether a difference in task difficulty may have influenced our results. However, we found no differences between the consolidation of visuospatial sequence knowledge, irrespective of whether this knowledge was supported by eye movements or not. Although learning effects were generally larger in the EM condition, where RTs were overall higher, the absolute sizes of these effects are not the measures of consolidation by themselves. More specifically, consolidation is derived from the comparison of the learning effects before and after the time delay, thus taking the size of the initial learning effect into account. In the current study, the sequence learning effect decreased similarly over the 24 hour interval in both conditions, irrespective of the size of the initial learning effect. Accordingly, the observed effects were highly unlikely to be modulated by task difficulty.

However, although there was no difference between the retention of visuospatial knowledge with and without eye movements in the current study, the use of the sequence knowledge at the end of Session 2 proved not entirely alike. Reaction times of participants in the EM condition, but not those of participants in the NEM condition, increased when they encountered a second untrained block (Block 5) in Session 2. Since participants in both conditions were able to display sequence knowledge after the second session in an awareness test, the disappearance of the RT learning effect at the end of Session 2 in the NEM condition can probably not be attributed to a loss of knowledge itself. It rather seems that, although knowledge was still present, participants in the NEM condition were not able to express it in their reaction times. In a previous study [11], we have already found that the expression of visuospatial sequence knowledge without the presence of a corresponding motor sequence is much more dependent on other task characteristics, like the perceptual difficulty of a task, than the expression of sequence knowledge that is supported by a (manual) response component. Accordingly, we surmise that facing deviant information twice in one session reduced the reliance on sequence knowledge in the NEM condition, but not in the EM condition, where a reliable oculomotor response sequence was present.

Finally, it has to be pointed out that we did not control for two factors that may have influenced the consolidation process in the current study. First, we did not use sleep diaries to assess whether the sleep-wake cycle of participants prior to the experiment was normal, though sleep may play an important role in the consolidation of sequence knowledge [23], [27], [28], [29], [30]. Yet, because we did control for sleep in the night between the first and the second session of the experiment, we can infer that participants in both conditions did not differ in the total number of hours they slept during that night, their sleep quality and the extent to which they felt rested. Second, our sample consisted of a majority of females in both conditions. There is a possibility that our results were influenced by the menstrual cycle of our participants, which has been shown to affect the consolidation of explicit motor sequence knowledge [39]. It might therefore be useful to take the effect of sex and female hormones into account in future consolidation studies, and to investigate whether the effect of hormones on memory can be generalized to implicit sequence learning tasks.

To conclude, in the current study we found that visuospatial sequence knowledge was not fully consolidated after a period of 24 hours, irrespective of whether this knowledge was supported by oculomotor movements. We therefore postulate that not the presence of eye movements, but other task aspects like the presence of random noise is important for whether or not sequence-specific knowledge is fully preserved over time.
